# Notes and News

**Published:** 1896-11-14

**Authors:** 


### NOTES AND NEWS.

(COLLECTED AND COMMUNICATED.)

THE epidemic of Beri Beri at the Richmond Asylum, Dublin, shows no diminution. Ninety-six persons have been attacked, and six deaths have occurred.

THE number of new medical students entering the metropolitan medical schools shows a decrease of 20 per cent. Although there is also a falling off in the provinces, at the Universities it is very slight indeed.

IT is proposed to erect a new Hospital for Consumption for Liverpool. West Kirby has been named as a likely locality. £50,000 is required, and £16,000 has already been subscribed.

WE are informed that an anonymous donor has given £1,000 to the Metropolitan Convalescent Institution, and has arranged that a number of admission orders shall be at the disposal of the directors of the Gas Light and Coke Company and the South Metropolitan Gas Company for the use of their employés, their wives, and children.

THAT vexed question, the grievances of the Irish dispensary doctors, is once again prominently before the public, and the Chief Secretary for Ireland consented, at the request of the Dublin branch of the British Medical Association, to receive a deputation on the subject. Mr. Thomson, president of the branch, stated the case for the officers.

A REPORT has been circulated that the new Lord Mayor intended to celebrate the sixtieth year of her Majesty's reign by endeavouring to raise £1,000,000 of money for the purpose of freeing the metropolitan hospitals from debt. We have made inquires as to this report at the Mansion House, and are informed by the Lord Mayor that while the matter is one keenly appealing to his sympathy, he does not think it could be brought to a practical solution.

THE Lord Mayor will pr side at the festival dinner of University College Hospital in February. He will be accompanied by the Lady Mayoress.

THE Royal University of Ireland has conferred honorary degrees on Dr. More Madden and Sir Charles Cameron. Dr. Madden is Obstetric Physician and Gynæcologist to the Mater Misericordiæ Hospital and Consulting Physician to the Children's Hospital; while Sir Charles Cameron is well known as the energetic Medical Officer of Health for Dublin.

ALTHOUGH the plague at Bombay is now abating, active measures are being carried forward of a precautionary nature. So far, only isolated cases have appeared elsewhere, and these have all been traceable to the same source of infection, Bombay. The Government have appointed a committee of scientific men to investigate the plague in all its aspects, and to act with a view of discovering curative and preventive measures.

THE recent action on the part of the Liverpool Corporation illustrates the beneficent advance of sanitation. The Local Government Board have been asked to sanction the loan of a sum of money to enable the Corporation to purchase and demolish a most insanitary part of the city, and erect in place of the condemned buildings public baths and washhouses. In some of the condemned localities the death-rate has been terribly high, reaching in one instance 79·6 per 1,000.

IT is decided to extend the Ingham Infirmary, South Shields. At a large public meeting held recently at South Shields, it was announced that the necessary expenditure was estimated at £7,000, and towards this the family of the late Mr. John Readhead had intimated their desire to contribute £3,000, and £1,000 towards the cost of furnishing. Mr. Readhead's name is to be associated with the extension building. Many other handsome donations were announced, leaving a deficit of little more than £800 on the estimated cost.

THE half-yearly report, just issued, of Dr. Collingridge, Medical Officer of Health for the Port of London, shows the large amount of sanitary work which is being done on the river. During the first six months of the present year 13,634 vessels of all classes had been inspected by the sanitary officers. A larger number of cases of infectious diseases were dealt with than during any previous half-year. At the hospital of the authority 37 patients were admitted, and two died; and the disinfecting apparatus was in use on 60 days for the disinfection of 9,634 articles, many of which, however, were disinfected for the Gravesend Corporation by arrangement.

THE removal of the patients from the old infirmary to the new Royal Halifax Infirmary was successfully accomplished last week. It was an undertaking requiring much forethought and care, for it was necessary that the removal of patients, nurses, and servants should all be accomplished in one day. By careful organisation on the part of the house surgeons and matron this was satisfactorily carried out. Five ambulances were employed, Dr. Maxwell, the house surgeon, seeing to the patients being comfortably placed in them, and Mr. Hunt, the assistant house surgeon, attending to the patients on their arrival at the new infirmary. The number of patients had been reduced as far as practicable, fifty patients being removed in the ambulance during the day. All patients, whether "in" or "out" patients, will now be treated at the new infirmary.

